# A Comprehensive Review of the Neglected and Emerging Oropouche Virus

**DOI:** 10.3390/v17030439

**Published:** 2025-03-19

**Authors:** Fengwei Bai, Prince M. D. Denyoh, Cassandra Urquhart, Sabin Shrestha, Donald A. Yee

**Affiliations:** School of Biological, Environmental, and Earth Sciences, The University of Southern Mississippi, Hattiesburg, MS 39406, USA

**Keywords:** Oropouche virus, biting midge, host factors, pathogenesis

## Abstract

Oropouche virus (OROV) is a neglected and emerging arbovirus that infects humans and animals in South and Central America. OROV is primarily transmitted to humans through the bites of infected midges and possibly some mosquitoes. It is the causative agent of Oropouche fever, which has high morbidity but low mortality rates in humans. The disease manifests in humans as high fever, headache, myalgia, arthralgia, photophobia, and, in some cases, meningitis and encephalitis. Additionally, a recent report suggests that OROV may cause fetal death, miscarriage, and microcephaly in newborns when women are infected during pregnancy, similar to the issues caused by the Zika virus (ZIKV), another mosquito-borne disease in the same regions. OROV was first reported in the mid-20th century in the Amazon basin. Since then, over 30 epidemics and more than 500,000 infection cases have been reported. The actual case numbers may be much higher due to frequent misdiagnosis, as OROV infection presents similar clinical symptoms to other co-circulating viruses, such as dengue virus (DENV), chikungunya virus (CHIKV), ZIKV, and West Nile virus (WNV). Due to climate change, increased travel, and urbanization, OROV infections have occurred at an increasing pace and have spread to new regions, with the potential to reach North America. According to the World Health Organization (WHO), over 10,000 cases were reported in 2024, including in areas where it was not previously detected. There is an urgent need to develop vaccines, antivirals, and specific diagnostic tools for OROV diseases. However, little is known about this surging virus, and no specific treatments or vaccines are available. In this article, we review the most recent progress in understanding virology, transmission, pathogenesis, diagnosis, host–vector dynamics, and antiviral vaccine development for OROV, and provide implications for future research directions.

## 1. Oropouche Virus

**Figure 1:**
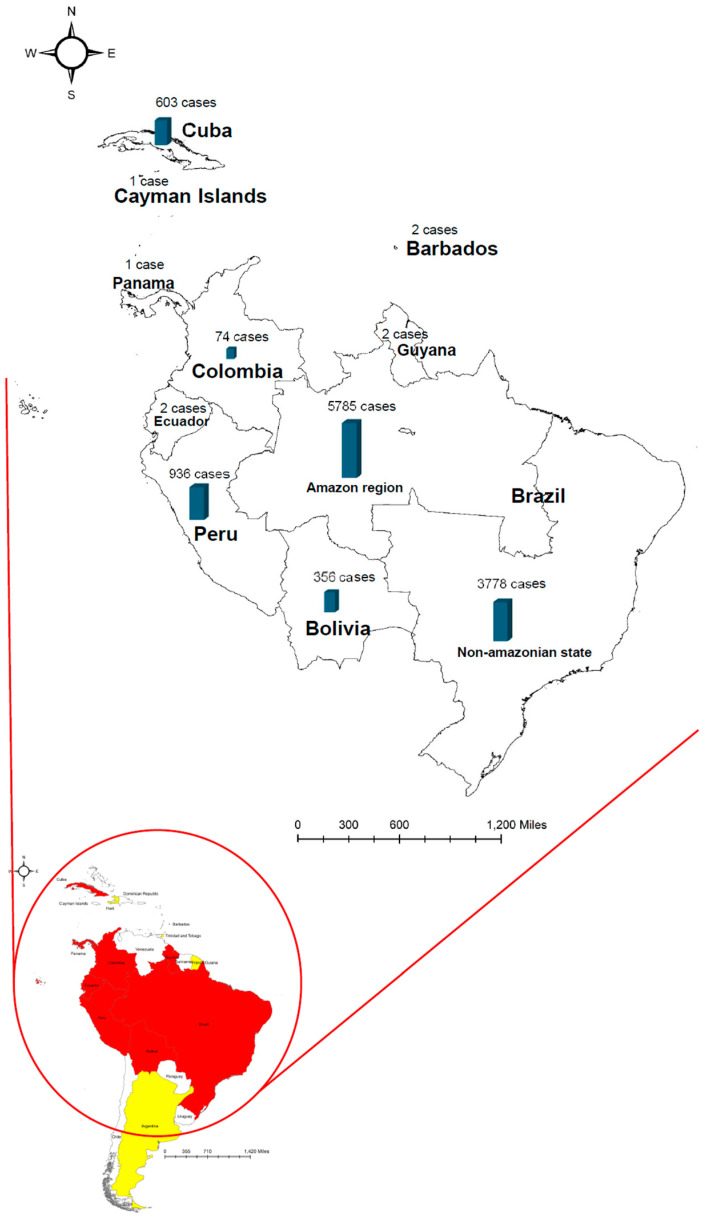
Human OROV cases reported before and in 2024 in the South and Central America regions. The yellow areas show the cases reported before 2023, including the outbreaks in Argentina, French Guiana, Haiti, and Trinidad and Tobago. The red areas show the cases reported in 2024. As of 25 November 2024, confirmed OROV cases were reported from the Amazon region of Brazil (5785 cases, illustrated with a blue column), the non-Amazon region of Brazil (3778 cases), and Bolivia (356 cases), Colombia (74 cases), Ecuador (2 cases), Peru (936 cases), Cuba (603 cases), Guyana (2 cases), Barbados (2 cases), and Panama (1 case). Data were retrieved from the WHO [3].

**Figure 2:**
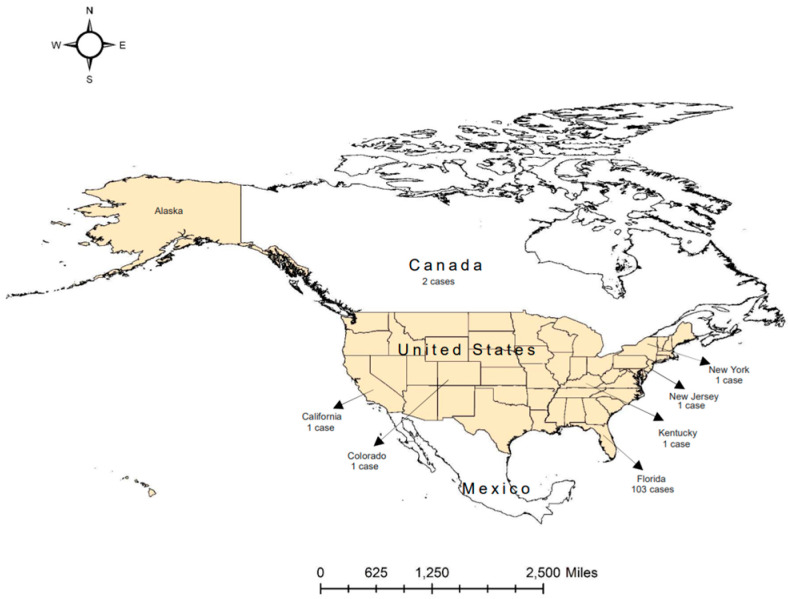
Travel-related OROV transmission to the US and Canada in 2024. A total of 110 OROV cases were reported in the US (108 cases) and Canada (2 cases), with a travel history to Cuba. Among 108 cases reported from the six states of the US, two were neuroinvasive, and the others were non-neuroinvasive. Data were retrieved from the US CDC [5].

Oropouche virus (OROV) was first isolated from the blood of a febrile charcoal worker near the Oropouche River in Trinidad and Tobago, West Indies, in 1955 [1,2]. Since then, at least 30 epidemics and more than 500,000 infection cases have been reported in South and Central America, specifically in the Amazon region. However, due to frequent misdiagnosis with other co-circulating viruses, such as dengue virus (DENV), chikungunya virus (CHIKV), Zika virus (ZIKV), and West Nile virus (WNV), that share similar clinical symptoms with OROV infection, the actual OROV case numbers may be much higher. Because of climate change, increased travel, and urbanization, OROV has recently infected more people at an increasing rate and has spread to new regions, potentially reaching North America. According to the World Health Organization (WHO), over 10,000 cases were reported in 2024, including in areas where it was not previously detected, including Barbados, Guyana, and Cuba (Figure 1). The first reported outbreak of OROV infection in Cuba occurred in May 2024 [3]. Importantly, OROV has also reached the United States (US), Canada, and Italy through travel [4]. According to the US Centers for Disease Control and Prevention (CDC), there were 108 OROV infection cases in the US in 2024, with the cases having a travel history to Cuba [5,6] (Figure 2). The WHO assesses the overall public health risk posed by OROV to be high at the regional level and low at the global level [3].

**Figure 3:**
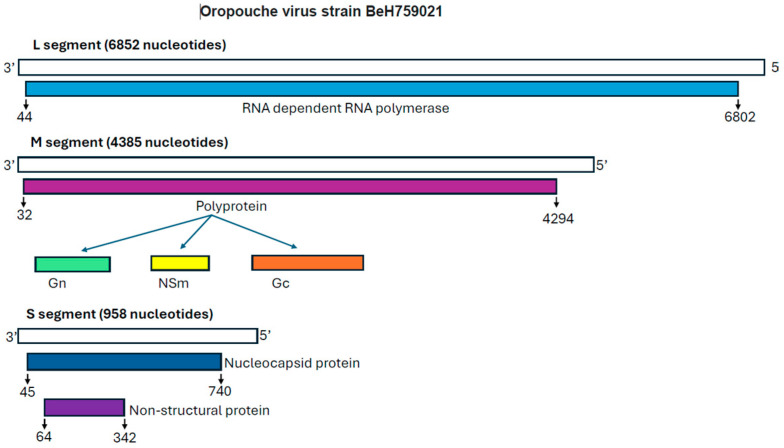
A schematic diagram showing the genome structure and the coded proteins of OROV. The diagram was drawn based on the sequence of the isolate BeH759021. GenBank Accession: L segment: KP691606.1, M segment: KP691607.1, and S segment: KP691608.1.

OROV belongs to the family *Peribunyaviridae* in the genus of *Orthobunyavirus* (OBV), the largest genus of RNA viruses with over 170 named viruses corresponding to 18 serogroups and 48 species complexes [2]. The viral particles of OBV are spherical, with a diameter ranging from 80 to 120 nm, with RNA segments encapsulated within a ribonucleocapsid (RNP) and wrapped within a bilayer lipid membrane acquired from the host cells through budding. Although high-resolution structures of OROV have not yet been determined, studies with other closely related OBVs suggest that the enveloped OROV virion is about 90 nm in diameter, displaying Gc and Gn glycoproteins on the surface [7]. OBVs have a negative sense, single-stranded RNA genome, which is segmented into Large (L), Medium (M), and Small (S) segments and gives rise to six structural and non-structural proteins [8] (Figure 3). The OROV L segment is about 6852 nucleotides long [9] and codes for the L protein, an RNA-dependent RNA polymerase (RdRp), which catalyzes the synthesis of the viral genomic RNA and transcribes the viral RNA genome into mRNA, which is then translated into viral proteins by the host cellular machinery. Because OBVs possess a negative sensed genome, which is not readily translated to make RdRp and other viral proteins once they enter host cells like positive sensed RNA viruses, they need to carry RdRp in the virions. The M segment has 4385 nucleotides [9] and encodes a polyprotein precursor that cleaves into two structural glycoproteins (Gn and Gc) and a nonstructural (NS) protein, NSm, which is encoded in between Gn and Gc [10,11]. Gn is a relatively small protein with an ectodomain of about 200 amino acids (aa) with one putative N-linked glycosylation site. In contrast, Gc, with an ectodomain of about 900 aa, is predicted to be a class II membrane fusion protein with 3 to 4 putative N-linked glycosylation sites [7]. Gn and Gc are produced in the ER lumen of infected cells, forming heterodimers that are transported to the Golgi apparatus, where new virions assemble and bud. These glycoproteins assemble into unique tripod-like spiky structures on the surface of the OBVs, which is crucial for the virus to attach to and enter host cells. Low-resolution electron cryo-tomography studies on the Bunyamwera virus (BUNV, a sister virus of OROV of the OBV genus) have shown that OBV particles feature prominent trimeric spikes by Gn and Gc [12]. An X-ray crystallography study of the N-terminal half of the Gc of four different OBVs, i.e., OROV, Schmallenberg virus (SBV), La Crosse Virus (LACV), and BUNV, showed that the spikes are formed by an N-terminal extension of the fusion glycoprotein Gc and that the projecting spike is the primary target of the neutralizing antibody response. Mice immunized with the spike domains elicit virtually sterilizing immunity [13]. The roles of NSm protein are still unclear, but they are believed to participate in the assembly and budding process of the progeny viruses [11,14]. However, in a reverse genetic study, mutant OROV lacking the NSm protein displayed characteristics similar to the wild-type virus, suggesting that the NSm protein may be dispensable for virus replication in the mammalian and mosquito cell lines [11]. The S segment is 946 bases long [9] and encodes the nucleocapsid (N) protein and another NS protein, NSs. The NSs are translated from a downstream AUG site on the same mRNA transcript as the N protein [7,11,15,16]. The N proteins form viral capsids that encapsulate viral genome segments. Due to its abundance, the N protein plays a crucial role in replication and transcription, ensuring the stability of the viral genome [17]. On the contrary, the NSs’ proteins are not included in the newly formed virions; instead, they play essential roles in aiding in viral replication and evading the host’s innate immune responses by suppressing type I interferon response, especially by the nine C-terminal amino acids of OROV NSs [11,18].

Because the OROV genome consists of three segments, a viral genome reassortment may occur when the virus co-infects a host with different strains of other related OBVs. The reassortment can lead to new viral strains with new genetic combinations, potentially altering their pathogenicity, transmissibility, and ability to evade the immune system. Recent studies have shown that novel reassortant strains of OROV have emerged, contributing to the outbreaks across regions. These new strains have been associated with increased pathogenicity, severe outcomes, such as maternal–fetal infections and congenital malformations, and higher transmission rates [19,20].

## 2. OROV Replication in the Host Cell

OROV has been suggested to enter the host cell through receptor-mediated endocytosis. A study on OROV entry with HeLa cells indicated that the OROV entry into HeLa cells was inhibited by chlorpromazine, an agent that blocks clathrin-dependent endocytosis in a dose-dependent manner [21]. Although a specific cellular receptor for OROV has not been identified, studies showed that the low-density lipoprotein-related protein 1 (Lrp1) efficiently mediated the viral cellular infection. Treating cells with either the high-affinity Lrp1 ligand receptor-associated protein or recombinant ectodomain truncations of Lrp1 significantly reduced OROV infection, suggesting Lrp1 is an indispensable host factor for the viral entry [22]. Interestingly, Lrp1 has also been shown to be an entry factor interacting with the Gn of the Rift Valley fever virus (RVFV), highlighting a broader role of Lrp1 in mediating the host-cell entry of diverse bunyaviruses [22,23]. The viral uncoating involves a fusion process of the envelope membrane and the endosome membrane triggered by an acidic environment within the late endosome, and the viral RNA is released into the cytoplasm. The study on HeLa cells showed that the OROV uncoating process was blocked by endosomal acidification inhibitors, further suggesting that the entry and uncoating of OROV occurs via a receptor-mediated endocytosis mechanism [21].

Once inside the host-cell cytoplasm, the negative-sense viral RNA genome can be converted to complementary positive antigenomic RNA segments for viral genome replication and protein translation mediated by the packaged RdRp in the virion. Unlike positive-sense RNA viruses, which can be directly translated by the host ribosomes, negative-sense RNA viruses need to be converted into positive-sense RNA by RdRp before translation can occur. Carrying RdRp in the OROV virions allows the virus to transcribe its RNA into mRNA upon entry into the host cell, facilitating rapid replication and protein synthesis. Despite lacking specific studies, OROV is predicted to use a “cap snatching” mechanism to initiate mRNA transcription because it is a common strategy shared by OBVs, such as RVFV [24]. After the viral RdRp or N binds to the 5′ cap structure of host mRNA, the viral endonuclease, a component of the RdRp, cleaves the host mRNA a few nucleotides downstream of the cap. The snatched capped RNA fragment is then used as a primer to initiate the synthesis of viral mRNA, which can then be translated by the host ribosomes. In addition, RdRp is also responsible for replicating the OBV genome by using the anti-genomic segments as templates [25]. The newly synthesized viral segments are wrapped with N proteins, forming nucleocapsids and assembling progeny virions, and both processes are believed to occur in the Golgi complex. The encapsulation by the N protein forms a ribonucleoprotein complex that associates both the RdRp and the surface glycoproteins Gn and Gc [18]. The two glycoproteins are integral membrane proteins with N-terminal ectodomains, and they associate with the host endoplasmic reticulum (ER) before being transported to the Golgi complex. The disruption of Golgi function using brefeldin A and monensin inhibits glycoprotein secretion [26]. OROV attracts the cellular endosomal sorting complexes required for transporting (ESCRT) machinery components to Golgi cisternae to mediate the membrane-remodeling events necessary for viral assembly and budding [27]. The immunoprecipitation and fluorescence microscopy experiments by Barbosa et al. have shown that ESCRT subunits, such as CHMP6, are recruited to the Golgi upon OROV glycoprotein expression [26]. Further, the small GTPase Rab27a has been shown to mediate the intracellular transport of OROV-induced compartments and viral release from infected cells, indicating that OROV hijacks Rab27a activity for intracellular transport and effective release from host cells [28].

## 3. Viral Transmission

**Figure 4:**
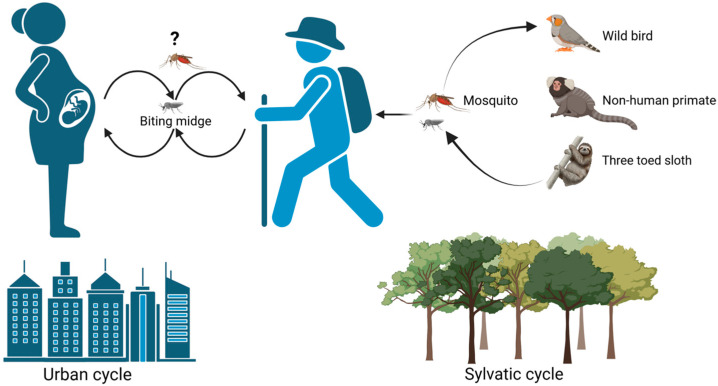
The sylvatic cycle and the urban cycle of OROV transmission. In the sylvatic cycle, potential reservoirs of the OROV include mammals such as sloths, non-human primates, and birds. The virus is maintained in nature between wild animals and forest-dwelling mosquitoes, particularly *Culex* species and the biting midge *Culicoides paraensis*. Infected humans from the sylvatic cycle can introduce the virus to urban areas, bridging the two transmission cycles. In the urban cycle, *Cu. paraensis* midges are the primary vector, and *Cx. quinquefasciatus* may also transmit OROV to humans. Besides arthropod vectors, human-to-human transmission may occur via mother-to-fetus, blood transfusion, and sexual transmission routes. The illustration was created in BioRender.com.

OROV is primarily transmitted to humans via the bite of infected *Culicoides (Cu.) paraensis* biting midges. These arthropods are abundant in tropical environments, making them ideal vectors for the virus. *Culicoides paraensis* prefers damp habitats, such as those that occur in the Amazon jungle and metropolitan areas with inadequate sanitation, where stagnant water allows for its reproduction. The OROV transmission cycle consists of a sylvatic cycle and an urban cycle (Figure 4). In the sylvatic cycle, the potential reservoirs of the OROV are mammals, non-human primates, and wild and some domestic birds [8,11,29]. In the sylvatic cycle, the virus is maintained in nature between wild animals, such as sloths and forest-dwelling mosquitoes, particularly the *Culex (Cx.)* genus. Even though OROV antibodies have been detected in wild mammals, implicating them as a potential reservoir for the OROV, more data are needed to support this notion [1]. Deforestation and changes in land use, such as agriculture and mining, can bring humans closer to the virus and its vectors. Humans have been suspected to be the link between the sylvatic cycle and the urban cycle of viral transmission of Oropouche fever. The virus is transmitted from person to person through the bites of infected midges. OROV replication generates a high titer in the blood, which is sufficient for naïve midges to pick up the virus from a viremic blood meal and become infected [30]. Although *Cu. paraensis* is the primary vector in the urban cycle [1], the mosquito *Cx. quinquefasciatus* has also demonstrated the potential for the infection, dissemination, and transmission of OROV, although at relatively low rates [31]. Humans are the only vertebrates observed to be involved in the urban cycle, and relevant studies have also ruled out the case of domestic animals transmitting the virus [2]. Although domestic animals, such as chickens, have been proposed as amplifiers, there is a lack of quantifiable data to confirm this concept [32]. Besides arthropod transmission, OROV can be transmitted vertically from mother to fetus and cause adverse pregnancy outcomes, such as fetal death and microcephaly [33,34,35], which is similar to other closely related OBVs in the Simbu serogroup that have been responsible for widespread epidemics of congenital disease in ruminants [36]. Although no cases of blood-transfusion transmission of OROV have been reported, similar to ZIKV and WNV transmission, it is possible that this virus can be transmitted from human to human without the arthropod vectors. Moreover, a recent report described that replication-competent OROV was detected in the semen of a patient, which raises concern about the possible risk of sexual transmission [37], similar to that of ZIKV [38,39]. However, no cases of sexual transmission of OROV have been reported [30].

## 4. Midge and Mosquito Vectors

Mosquitoes and midges are among the most significant vectors of arboviruses transmitting numerous viral pathogens that significantly affect human and animal health. Insects within the family Culicidae (mosquitoes) and Ceratopogonidae biting midges are implicated in transmitting OROV. For the former, *Aedes serratus*, *Cx. quinquefasciatus*, *Coquillettidia venezuelensis*, and *Mansonia venezuelensis* have been captured in the wild, infected with OROV [40]. In addition, laboratory infection has been accomplished for *Ae. serratus*, *Ae. scapularis*, *Ae. albopictus*, *Cx. quinquefasciatus*, *Cq. venezuelensis*, and *Psorophora ferox* [41,42,43,44,45]; *Cx. tarsalis* is unlikely to be a competent vector, whereas *Cx. quinquefasciatus* appears to be the most competent vector of those tested in the laboratory [42,46]. Despite evidence of viral infection in mosquitoes, the biting midge *Cu. paraensis* is considered to be primarily responsible for OROV transmission [45,46,47]. Adult *Cu. paraensis* midges have been shown to transmit OROV in the laboratory [47], and OROV has been isolated from field-collected adults [48]. *Culicoides sonorensis*, a species common to North America, shows a high rate of infection, dissemination, and transmission of OROV in the lab [42]. Other species of *Culicoides* could also be competent for OROV transmission, but to date, none have been tested.

*Culicoides paraensis* belongs to the family *Ceratopogonidae*, which includes 125 genera and around 5500 species. Four of these genera are known to possess species that suck vertebrate blood, which include *Austroconops*, *Culicoides*, *Forcipomyia* subgenus *Lasiohelea*, and *Leptoconops*. *Culicoides* can be easily distinguished from others by their wing characteristics and are widely distributed worldwide, except Antarctica, being associated with transmitting multiple pathogens of medical and veterinary importance. Biological transmission has been supported via *Cu. paraensis* from infected to susceptible hamsters and infected people to susceptible hamsters, with transfer rates of up to 83% [1,49]. Besides OROV, the biting midges also transmit other viruses, such as bluetongue virus (BTV), epizootic hemorrhagic disease virus (EHDV), Akabane virus (AKAV), bovine ephemeral fever virus (BEFV), and African horse-sickness virus (AHSV) [50,51,52].

The distribution of *Cu. paraensis* ranges from Argentina to parts of the northern US, and *Cu. sonorensis* spreads from parts of Mexico, the US, and Canada. There are over 1300 species of *Culicoides*, and at least 13% are unclassified [1,53]. Most (96%) of *Culicoides* are hematophagous, with females taking blood meals to facilitate egg production [1,53]. The life cycle is holometabolous, with females depositing eggs either individually or in clusters in either aquatic habitats or semi-aquatic habitats, including on or near mud and animal waste [1,50,51]. The life cycle of *Cu. paraensis* consists of an egg stage, four larval stages, pupa, and imago, with a life span of 20–30 days. Larvae go through four instars, the last of which may overwinter in some species, although development typically takes a few weeks, with variations due to temperature and humidity [51,52]. Adult dispersal may include short flights and more passive movement on wind currents due to the small size of adults [1]. Although most *Culicoides* species have crepuscular biting habits [51,52,54], *Cu. paraensis*, the primary urban vector of OROV, has diurnal peaks in biting activity [51,55,56]. The larvae of this species develop in semi-aquatic habitats that remain moist during short dry periods, such as tree holes, rotting fruit, old stumps, and river banks [1,56]. Biting occurs outdoors in shaded areas and indoors, and increases during rainfall but decreases when conditions are hot and dry [51,55,56]. The use of rotting fruit waste as larval habitat, especially bananas, plantains, and cacao husks, provides opportunities for human and midge interaction in peri-urban areas [51,55,56].

## 5. Control Measures for Midges

Besides antiviral vaccines, the most effective option for limiting arboviral diseases is to control the vector populations. However, unlike other mosquito-borne diseases, OROV poses unique challenges in controlling its primary vector. Suppression and control techniques for mosquitoes, especially open-water species like *Cu. quinquefasciatus*, are diverse and well known. In contrast, most control approaches for *Culicoides* were developed for agricultural areas [42], which is problematic given the importance of the urban cycle of this disease. There are no specific chemical approaches for *Cu. paraensis*; however, insecticides, such as deltamethrin, and repellents like N, N-Diethyl-meta-toluamide (DEET), and plant-derived essential oils (eucalyptus, geraniol, neem), are known to be effective against *Culicoides* species in general [57,58]. A note of caution is that deltamethrin may only reduce, but not eliminate, the biting risk of *Culicoides*, especially when applied to livestock [59]. Besides chemical applications, another approach may involve the source reduction in juvenile habitats, especially manure and other substrates where the biting midges develop [59]. However, considering the breadth of both natural (e.g., stream banks) and human-produced larval habitats (e.g., food waste) for *Cu. paraensis*, the source-reduction approach is extremely challenging [53]. Finally, the education of the personnel who are most likely to encounter biting-midge vectors (e.g., farmers, ranchers, forest workers, and tourists) may also be effective as part of an integrated approach to reducing the prevalence of OROV [1].

## 6. Clinical Symptoms

Oropouche fever outbreaks tend to follow a seasonal pattern, with most cases occurring during the rainy season. After an infected biting midge or mosquito bite, the virus initially replicates in local cells, such as endothelial cells, epithelial cells, and macrophages. It then enters the bloodstream of the infected individuals, leading to viremia and manifesting OROV symptoms, such as high fever, headache, myalgia, and arthralgia [60]. These symptoms are not specific to OROV infection but are shared with other co-circulating arboviruses in the region, such as DENV, WNV, ZIKV, CHIKV, and even influenza [61]. OROV infections in golden hamsters showed that the virus was detected in high titers in blood, liver, and brain but not in the other organs. Histopathology revealed meningoencephalitis and hepatitis, with abundant OROV antigen detected in the liver and brain [62]. However, there is no substantial evidence to suggest that OROV causes liver diseases in humans, although some OROV fever patients have shown mildly elevated liver enzymes [30]. Some infected individuals present with less common symptoms, such as rash, retro-orbital pain, anorexia, and hemorrhagic manifestations [61,63,64]. Many patients tested during the first few days of clinical disease are viremic, with virus titers between 10^3^ and 10^7^ infectious doses per ml of serum, concentrations that can transmit the virus to naïve female midges through a blood meal [65]. This supports the view that humans are the sole amplifying host during urban outbreaks [30]. Besides blood, OROV was detected in the cerebrospinal fluid (CSF) in some neuroinvasive cases [33]. In one reported case, OROV RNA was detected in urine and saliva, which is typical for ZIKV. Infections in the central nervous system (CNS) that result in aseptic meningitis or meningoencephalitis are severe clinical manifestations of the virus, though this appears to be very rare [66,67,68,69]. CNS symptoms frequently occur in immunocompromised adults and children [70]. The OROV disease condition usually has two phases: an acute phase lasting 2–4 days, followed by remission, and a return of symptoms after 7–10 days [69]. Most patients recover without complications; however, chronic myalgia and asthenia lasting up to a month have also been documented [67]. Although deaths caused by OROV are very rare, the virus can lead to severe complications, including neuroinvasive diseases such as Guillain–Barré syndrome, meningitis, and encephalitis, which can be fatal [30]. In addition, there have been cases of adverse pregnancy outcomes, including fetal death or congenital abnormalities, such as microcephaly [71].

## 7. Diagnostics of OROV Infection

Diagnosing OROV is challenging due to its symptoms being similar to other viral diseases, such as DENV, ZIKV, and CHIKV, in the same areas. This has necessitated specific and reliable diagnostic methods to differentiate OROV from other febrile illnesses endemic to the regions. An advantage is that the peak viremia time of OROV infection usually coincides with the onset of acute febrile illness, and OROV viral RNA and antigens in the blood can be detected in the blood [7]. In addition, infected individuals have been found to produce IgM and IgG antibodies in serum and the cerebrospinal fluid within 2 weeks after onset, allowing for serological testing [72]. Although OROV RNA was detected in saliva and urine from a patient whose samples were collected five days after the onset of symptoms [73], suggesting possible non-invasive diagnostics, more studies must be conducted to determine the feasibility of this possibility, since only one out of five test patients was positive in the saliva and urine samples [73].

Molecular detection technologies, including real-time reverse-transcription quantitative PCR (RT-qPCR), are the gold standard for acute OROV diagnosis, showing great sensitivity and specificity. A modified one-step real-time RT-qPCR in sera taken within the first 5 days of sickness demonstrated a 93% detection rate [74]. To aid in the identification of both OROV and reassorted strains, RT-qPCR, RT-PCR, and nested RT-PCR technologies have been developed to target the virus’s M segment. However, these methods require more validation before being widely used in clinical studies [8]. Serological methods have been the go-to method for identifying human OROV-specific antibodies [1]. Several of these serological methods have been employed to diagnose OROV infections, of which ELISA is one of the most used serological methods, enabling the detection of OROV-specific IgM and IgG antibodies. IgM and IgG ELISAs have been successfully used to differentiate OROV from other arboviruses [75]. Some studies have suggested that IgM testing in CSF should be conducted concurrently with IgM testing in matched serum samples and supported by studies of blood–brain barrier permeability to confirm intrathecal immunoglobulin production [76]. However, there are few commercially available ELISA kits for OROV; hence, most investigations rely on in-house tests with recombinant antigens. Cross-reactivity with antibodies from other OBVs is also a barrier to testing the assay’s specificity. Moreover, the hemagglutinin inhibition test has been widely employed in serological surveys because it can identify antibodies long after natural infection, and it has been used to screen for 19 different arboviruses [77]. Compared to other serological tests, it has a good sensitivity but low specificity, often resulting in cross-reactions [78]. Another useful diagnostic tool that has been developed and used to diagnose OROV is an immunofluorescence test using peripheral white blood cells from patients [79]. A far less used approach for diagnosing OROV is metagenomics, when other RT-qPCR systems fail to detect viral OROV RNA [80]. The metagenomic examination of patient samples has been used to discover and identify divergent strains of OROV. However, the drawback to using this method is that metagenomic tools and facilities are not generally available in arbovirus-infected areas.

## 8. Pathogenesis

Despite its serious concerns about mortality and potential fast spread to new regions, limited knowledge is available on the pathogenesis of OROV. Studies reveal that adult immunocompetent wild-type (WT) mice usually resist peripheral OROV infection. OROV infections have been investigated in golden hamsters (*Mesocricetus auratus*) and neonatal BALB/c mice. The virus was detected in the brain and spinal cord, associated with pathological evidence of encephalitis, and resulted in lethality [62,81]. Knockout mice with targeted immune-system impairments, especially interferon (IFN)-related pathways, are prone to OROV infection and often experience lethal outcomes, indicating the critical roles of innate immunity in controlling OROV infection in vivo. Innate immune responses serve as the first line of defense to constrain virus replication. Non-myeloid cells, such as fibroblasts and dendritic cells, are essential in the early defense against OROV infection [82]. Upon viral infections, the host-cellular intrinsic components, such as pattern-recognition receptors (PRRs), recognize viral pathogen-associated molecular patterns (PAMPs), activating signaling cascades that produce various chemokines and cytokines. The OROV genome, serving as an important PAMP, can be sensed by Toll-like receptors and RIG-like receptors, triggering inflammatory and antiviral cytokine responses. 

Type I interferons (IFNs), including IFN-α and -β, are activated at the early stages of virus replication and play an essential role in inhibiting viral replication by triggering the expression of antiviral interferon-stimulated genes (ISGs). The innate immune responses induced by OROV infection are mediated by the induction of a type I IFN pathway through Interferon Regulatory Factors (IRF3, IRF5, and IRF7), as well as the interferon-α and -β receptors, and Mitochondrial Antiviral Signaling Protein (MAVS) in the CNS infection of OBVs, including OROV, in the mouse model [83]. Compared to IRF3 and IRF7, IRF5 has been demonstrated to play a major role in modulating the host-type I IFN responses in the peripheral organs that control OROV and LACV, a closely related encephalitic OBV infection, neuroinvasion, in mice [83]. In another study of cellular and mouse models of OROV and LCMV, MAVS, IRF-3, IRF-7, and the receptor for type I IFN signaling (IFNAR) have been reported to restrict OROV replication and liver injury. In bone-marrow chimera studies, recipient-irradiated *Ifnar^-/-^* mice reconstituted with WT hematopoietic cells sustained high levels of OROV replication and liver damage, whereas WT mice reconstituted with *Ifnar^-/-^* bone marrow were resistant to disease, suggesting that IFN signaling in non-myeloid cells contributes to the host defense against OBVs [82]. 

*In vitro* OROV infection in human PBMCs significantly increases the mRNA expression of type I IFN-α and -β, and the ISGs, i.e., Mx1 and IFIT1 [84]. However, studies of infections of negative-sense RNA viruses of the *Orthomyxoviridae*, *Bunyaviridae*, and *Filoviridae* families with *Ifit1^-/-^* mice and the primary cells and human alveolar basal epithelial cells, A549 cells suggested that IFIT1 is not a dominant restriction factor against negative-sense RNA viruses, including OROV [85]. In addition, the expression of the RIG-I-like receptor dsRNA helicase MDA5 and the TLR3 adapter molecule TRIF significantly increases at the mRNA level. Moreover, the blockage of type I IFN receptor (IFNAR) with anti-IFNAR antibody enhances OROV replication in the PBMCs [84]. Interestingly, OROV may persist in different types of human leukocytes and be reactivated after blocking the IFN pathway in specific microenvironments during events of immunosuppression [84]. These results demonstrate the importance of the type I IFN response to OROV infection in the peripheral blood. Moreover, the experiments with type I IFN KO mice indicated that OROV-infected *Ifn-β^-/-^* mice exhibited a lethality rate of 17%, suggesting that IFN-β plays some role in protection against OROV infection in mice. In contrast, IFN-α/β receptor KO (*Ifnar^-/-^*) mice exhibited 100% lethality, suggesting that IFN-α may be critical in controlling OROV infection [82]. Consistent with this finding, IFN-α production was consistently high in the serum of the acute OROV fever patients throughout the timeline kinetics in both early and late seroconversion when compared to the healthy controls, suggesting it is a universal biomarker of human acute-phase OROV fever [72]. Moreover, in vitro experiments have shown that IFN-β mRNA levels increase in the first hour and drop rapidly, reaching very low levels at 24 h post-OROV infection [83]. Besides type I IFN, the mRNA of type III IFN, which also has a direct antiviral role against viral infections, significantly increased, while type II IFN (IFN-γ) increased in PBMC at 24 h and slightly decreased 48 h post-OROV infection [84].

Type II interferon, interferon-γ (IFN-γ), is a pleiotropic cytokine-modulating innate and adaptive immune networks by stimulating adaptive antigen-specific immunity and triggering innate cell-mediated immunity, particularly through activating macrophages. Unlike type I IFN, which can be produced by virtually all types of cells upon the detection of viral or microbial invasion, IFN-γ is produced by antigen-activated T lymphocytes and cytokine-activated group 1 innate lymphoid cells (ILC1). In addition, due to its broad immunomodulatory activities, IFN-γ lacks a specific antiviral mechanism. Its antiviral functions range from establishing an antiviral state and coordinating immune responses for the long-term control of viral infection; activating tissue-resident dendritic cells, macrophages, and NK cells for augmented inflammation and antiviral functions; and modulating the differentiation and maturation of T cells and B cells, to inducing type I IFN production [86]. A low level of IFN-γ production was detected in the serum of the OROV fever patients, suggesting its immunomodulating role in the pathogenesis of OROV [72].

Although the majority of OROV infections cause Oropouche fever symptoms, neurological infections have also been documented in some patients [1,66,69]. OROV was isolated in Vero cell culture from the CSF of an infected individual, and the OROV RNA was detected in the CSF from patients [66], indicating that OROV can infect the central nervous system (CNS). Using neonatal BALB/c mice inoculated with OROV by the subcutaneous route, a study revealed the progression of OROV in the CNS by spreading into the posterior parts of the brain, including the periaqueductal gray, toward the forebrain. In the early phases of the infection, OROV gains access to neural routes, reaching the spinal cord and ascending to the brain, presumably through retrograde axonal transport [33]. Later, OROV crosses the blood–brain barrier, resulting in a more intense spread into the brain parenchyma, with more severe manifestations of encephalitis [87]. Another study using adult human-brain slices of OROV infection suggests that human neural cells and microglia, but not astrocytes, can be infected ex vivo by OROV [33]. In addition, OROV infection led to the release of the pro-inflammatory cytokine tumor necrosis factor-alpha (TNF-α) and induced neurodegeneration, indicating that OROV triggers an inflammatory response and tissue damage [33]. Further, it has been shown that OROV infects human peripheral blood mononuclear cells (PBMC), but the infection was not productive, since neither antigenome nor infectious particle was found in the supernatant of infected PBMCs. Interestingly, OROV was able to infect and remain in low titers in human T cells, monocytes, DCs, and B cells, indicating the possibility of leukocytes serving as a trojan horse to transport OROV to the CNS [84]; however, future studies are warranted to dissect the detailed mechanisms by which OROV enters the CNS.

OROV has been documented to cause adverse pregnancy outcomes, such as fetal death and microcephaly [33,34,35]. Recent reports indicate that OROV infection during the first trimester of pregnancy might lead to severe outcomes, including fetal death or congenital abnormalities, similar to ZIKV infection during pregnancy [88,89]. A clinical study in Espírito Santo State, Brazil, 2024 reported OROV fever in pregnant women and their neonates [90]. Of fifteen pregnancies, two infections occurred in the first trimester, resulting in one spontaneous abortion and one live birth with corpus callosum dysgenesis, with RT-PCR positive for OROV RNA in the neonate’s serum sample one day after birth, indicating vertical mother–fetal transmission. In contrast, the remaining thirteen infections occurred during the third trimester, leading to no anomalies in the neonates at birth, with one showing a possible intrapartum transmission of the virus during cesarean section. Interestingly, five pregnant women infected with OROV in the third trimester had positive RT-PCR for OROV RNA in the placenta fragment but not their neonates [90], implying OROV may not efficiently pass through the blood–placenta barrier in the late stage of the pregnancy. The transplacental transmission of OROV is likely dependent on an episode of maternal viremia, a condition often observed in viral vertical transmissions, including those caused by other OBVs and ZIKV [71,91,92]. Early-stage congenital infection often results in more severe adverse outcomes, including miscarriage, stillbirth, and microcephaly. OROV RNA has been detected in multiple organs in stillbirth fetuses, including blood, spleen, liver, kidneys, lungs, heart, and brain [92]. A histopathological analysis of autopsy samples of an infant with congenital OROV infection showed the necrosis of neurons, microglia, and astrocytes with viral antigens detected in multiple organs [92]. These clinical reports highlight the urgent need for effective measures for preventing and managing OROV infection in pregnant women. Additionally, congenital OROV infection may follow the patterns of other viral infections during pregnancy, such as ZIKV, cytomegalovirus (CMV), and rubella, which cause postnatal manifestations. Some healthy newborn babies can still develop postnatal congenital ZIKV syndromes, including head-growth restrictions and behavioral deficits, before 1 year of age [89,93,94]. Therefore, it is necessary to closely monitor both physical and intellectual development in children whose mothers were exposed to OROV infection during pregnancy. Furthermore, animal models are needed to study the mechanism of OROV transplacental transmission and congenital infection.

## 9. Vaccine and Antiviral Development

Currently, there are no approved vaccines for Oropouche fever, and developing vaccines and antivirals are urgently needed. Several strategies have been considered when developing vaccines against OROV. These strategies include live attenuated, chemically inactivated, DNA-vectored, and protein-subunit immunization strategies [53]. Most notable among the strategies that have been studied thus far is a candidate vaccine that is based on a replication-competent chimeric vesicular stomatitis virus (VSV), which was created by replacing the indigenous glycoprotein with the OROV glycoprotein complex (GPC) [95]. Sera collected from challenged mice showed that the vaccine candidate generated neutralizing immunity against wild-type OROV and reduced the viral load and disease severity. Another strategy involves using immunoinformatics to identify epitope-based vaccine candidates and ligand-binding pockets for making epitope-based peptide vaccines against OROV [96]. Another strategy for developing OROV vaccines involves using vaccines against other OBVs as guides to making OROV vaccines, such as those against the Schmallenberg virus [97], Aino virus, and Akabane virus [98]. In Europe, these vaccines have been approved for use in sheep and cattle because these viruses are the major causes of veterinary infection among livestock [99]. In a study, two truncated recombinant AKAV Gc proteins were expressed using the *E. coli* expression system to identify the epitope for viral neutralization. The study showed that these epitopes showed good neutralizing activity when mice were immunized with the recombinant proteins [100]. IFNAR^-/-^ mice were challenged with two deletion mutants of Schmallenberg virus, which lack the nonstructural protein NSs or NSm. The results show that mice challenged with this vaccine had low viremia and no weight change [101].

Research is ongoing to identify potential therapeutic targets by understanding the host–virus interactions. For instance, the host protein low-density lipoprotein-related protein 1 (Lrp1) has been identified as crucial for OROV and RVFV cellular entry, suggesting it could be a target for developing pan-bunyaviral therapeutics [22,23]. Small GTPase Rab27a mediates the intracellular transport of OROV-induced compartments and viral release from host cells, and depleting Myosin Va, a downstream effector of Rab27a, or inhibiting actin polymerization inhibited OROV egress, suggesting potential targets for therapeutic interventions for Oropouche fever. Cellular miRNAs are short, non-coding RNAs that post-transcriptionally regulate gene expression and play key roles in viral replication. miR-217 or miR-576-3p have been shown to promote OROV replication in human hepatocarcinoma cell line HuH-7, and inhibiting these two miRNAs resulted in the restriction of OROV replication [102]. Acridone-based substances have demonstrated notable antiviral action against DNA viruses from the *Herpesviridae* family and RNA viruses from different families, primarily the *Flaviviridae* family. A study has shown that acridones FAC21 and FAC22 efficiently inhibited OROV replication by 99.9% in vitro by interacting with the OROV endonuclease [103]. The endonuclease of OBVs is the enzyme involved in cap-snatching activities, and it is a potential antiviral target inhibiting OROV replication. A study suggested that the compounds from hops (*Humulus lupulus* L.) beta acids, humulone, xanthohumol, flavonoids, and alpha acids hindered OROV replication at an early stage of the viral cycle from 12 to 48 h postinfection by interacting with the endonuclease [104]. In addition, a recent study showed the *in silico* effects of Wedelolactone (WDL) on the OROV endonuclease and its potential inhibitory effects on several steps of viral infection in mammalian cells *in vitro* [105].

## 10. Concluding Remarks

Despite its significant health risks, the virology and pathogenesis of OROV remain poorly understood. Limited information on this emerging virus has largely been gleaned from the related viruses. The overlapping symptoms with other arboviruses in tropical and developing countries make developing inexpensive, fast, specific, and sensitive detection methods from clinical samples incredibly challenging. Although the death rate from OROV infection is low, its ability to cause neuroinvasive diseases and congenital deficits in newborns is particularly concerning. Given its potential for evolution through mutations and genome reassortment, more pathogenic strains may emerge and spread to new areas, including North America. Although midges are likely to serve as the primary vector for humans, a more comprehensive understanding of the insects involved, including mosquitoes, is of high priority. In addition, urgent measures are needed to develop control strategies for the midge population in urban locations, and develop safe and effective vaccines and antivirals. With the rapid development of artificial intelligence (AI) and its application in drug design, more effective structure-based antiviral drugs are expected to target critical viral proteins, such as RdRP and the endonuclease of OBVs, including OROV. Future investigations are also warranted to understand how OROV invades the CNS, causing neuronal diseases, and how it crosses the placenta to infect neural progenitors in fetuses.
